# Human Monoclonal Antibodies Broadly Neutralizing against Influenza B Virus

**DOI:** 10.1371/journal.ppat.1003150

**Published:** 2013-02-07

**Authors:** Mayo Yasugi, Ritsuko Kubota-Koketsu, Akifumi Yamashita, Norihito Kawashita, Anariwa Du, Tadahiro Sasaki, Mitsuhiro Nishimura, Ryo Misaki, Motoki Kuhara, Naphatsawan Boonsathorn, Kazuhito Fujiyama, Yoshinobu Okuno, Takaaki Nakaya, Kazuyoshi Ikuta

**Affiliations:** 1 Department of Virology, Research Institute for Microbial Diseases (RIMD), Osaka University, Suita, Osaka, Japan; 2 Graduate School of Life and Environmental Sciences, Osaka Prefecture University, Izumisano, Osaka, Japan; 3 The Japan Science and Technology Agency/Japan International Cooperation Agency, Science and Technology Research Partnership for Sustainable Development (JST/JICA, SATREPS), Tokyo, Japan; 4 Kanonji Institute, The Research Foundation for Microbial Diseases of Osaka University, Kanonji, Kagawa, Japan; 5 Department of Genome Informatics, RIMD, Osaka University, Suita, Osaka, Japan; 6 Department of Environmental Pharmacometrics, Graduate School of Pharmaceutical Sciences, Osaka University, Suita, Osaka, Japan; 7 Applied Microbiology Laboratory, International Center of Biotechnology, Osaka University, Suita, Osaka, Japan; 8 Ina Laboratory, Medical & Biological Laboratories Corporation, Ltd., Ina, Nagano, Japan; 9 Department of Medical Sciences, Ministry of Public Health, Muang, Nonthaburi, Thailand; 10 International Research Center for Infectious Diseases (RIMD), Osaka University, Suita, Osaka, Japan; Paul-Ehrlich-Institut, Germany

## Abstract

Influenza virus has the ability to evade host immune surveillance through rapid viral genetic drift and reassortment; therefore, it remains a continuous public health threat. The development of vaccines producing broadly reactive antibodies, as well as therapeutic strategies using human neutralizing monoclonal antibodies (HuMAbs) with global reactivity, has been gathering great interest recently. Here, three hybridoma clones producing HuMAbs against influenza B virus, designated 5A7, 3A2 and 10C4, were prepared using peripheral lymphocytes from vaccinated volunteers, and were investigated for broad cross-reactive neutralizing activity. Of these HuMAbs, 3A2 and 10C4, which recognize the readily mutable 190-helix region near the receptor binding site in the hemagglutinin (HA) protein, react only with the Yamagata lineage of influenza B virus. By contrast, HuMAb 5A7 broadly neutralizes influenza B strains that were isolated from 1985 to 2006, belonging to both Yamagata and Victoria lineages. Epitope mapping revealed that 5A7 recognizes 316G, 318C and 321W near the C terminal of HA1, a highly conserved region in influenza B virus. Indeed, no mutations in the amino acid residues of the epitope region were induced, even after the virus was passaged ten times in the presence of HuMAb 5A7. Moreover, 5A7 showed significant therapeutic efficacy in mice, even when it was administered 72 hours post-infection. These results indicate that 5A7 is a promising candidate for developing therapeutics, and provide insight for the development of a universal vaccine against influenza B virus.

## Author Summary

Influenza virus is classified into types A, B and C. Influenza A virus is further divided into many subtypes, all of which exist in animals, indicating pandemic potential. By contrast, influenza B virus circulates almost exclusively in humans and, as there is no evidence for reassortment with influenza A virus, there is no indication of pandemic potential. Hence, there is far less accumulated research information regarding influenza B virus than influenza A virus. Influenza B virus, which is classified into two phylogenetic lineages, does, however, cause annual epidemics in humans and is therefore as essential to control as influenza A virus. Recently, the development of a universal vaccine and therapeutic strategies using human monoclonal antibodies (HuMAbs) has been gathering great interest. The present study reports a HuMAb neutralizing a wide range of influenza B viruses of both lineages. This HuMAb recognizes the conserved region of hemagglutinin. Moreover, therapeutic efficacy of this HuMAb was also confirmed by *in vivo* animal experiments. Thus, this study provides insight for the development of broad-spectrum therapeutics and a universal prophylactic vaccine against influenza B virus.

## Introduction

Influenza virus remains a constant public health threat. During annual epidemics 5–15% of the worldwide population are typically infected, resulting in 3 to 5 million cases of severe illness and between 250,000 to 500,000 deaths per year [1], [2]. While all age groups are affected by the disease, most influenza-related hospitalizations in industrialized countries occur in young children, the elderly, and the immunocompromised [3]. Like H1 and H3 subtypes of influenza A virus, influenza B virus also causes epidemics in humans [2]. In contrast to influenza A virus, influenza B virus is found almost exclusively in humans and has a much slower mutational rate [3]–[5]. However, cocirculation of two phylogenetically and antigenically distinct lineages, represented by B/Yamagata/16/1988 and B/Victoria/2/1987, has caused antigenic variation through genetic reassortment and antigenic drift from cumulative mutations, leading to annual epidemics [6], [7].

Currently, two main countermeasures, vaccines and antiviral drugs, are used against influenza virus [8]. Vaccination has been the mainstay of infection control. However, the protection afforded by vaccination varies widely, depending on the antigenic match between the viral strains in the vaccine and those that are circulating during a given influenza season, as well as on the recipient's age and health status [1], [3], [9]. Neuraminidase inhibitors, such as oseltamivir (Tamiflu) and zanamivir (Relenza), and matrix 2 (M2) ion channel inhibitors like amantadine, have been widely used for the treatment of influenza viral infection and are proven to be quite effective against susceptible strains [10]. However, they have limited efficacy in delayed administration after the onset of illness [11], and widespread use has resulted in the emergence of resistant viral strains, as seen in H1N1 and H3N2 [12]–[15]. Of note, M2 blockers are not active against influenza B virus as it has no equivalent to the M2 ion channel protein of influenza A virus [16]. Thus, the development of therapeutic approaches and vaccine design that provide potent and broadly cross-protective host immunity are a global public health priority [17].

Human monoclonal antibodies (HuMAbs) prepared from vaccinated donors and patients with viral infections could have potential therapeutic application, and provide significant information on human epitopes that could be important for developing the next generation of universal vaccines [18], [19]. In addition to classical hybridoma methods [20], recent advances in technology, such as transgenic mice [21] and yeast or phage display [22], [23], have renewed interest in the development of HuMAbs [17], [24], [25]. Thus, using phage display or single cell culture methods, several HuMAbs with broad neutralizing activities have been identified against the hemagglutinin (HA) protein in influenza A viruses, including C6261 and F10 which react with group 1 [26], [27], and CR8020 which reacts with group 2 viruses [28]. Another HuMAb, FI6v3, that neutralizes both group 1 and group 2 influenza A viruses, has also recently been described [29]. For influenza B virus, by contrast, broadly neutralizing HuMAbs, CR8033, CR8071 and CR9114, have firstly reported on September 2012 [30].

A hybridoma method for establishing HuMAbs was developed previously in this laboratory by fusion of the peripheral blood mononuclear cells (PBMCs) of influenza-vaccinated healthy volunteers with the fusion partner cell line SPYMEG, which has been optimized for higher reliability of cell fusion by overcoming chromosome deletion problem [31], [32]. In this study, three HuMAbs that neutralize influenza B virus were prepared using this method. One of the three HuMAbs, 5A7, reacted broadly with influenza B virus isolates from 1985 to 2006 that belong to both the Yamagata and Victoria lineages, and recognized a highly conserved region in the HA protein. Moreover, 5A7 showed therapeutic efficacy even in mice treated with the HuMAb 72 hours post-infection. These results indicate that 5A7 is a promising candidate for developing therapeutics and will provide insight for the development of the next generation of vaccines universally effective against influenza B virus.

## Results

### Preparation of anti-influenza B HuMAbs

**Figure 1:**
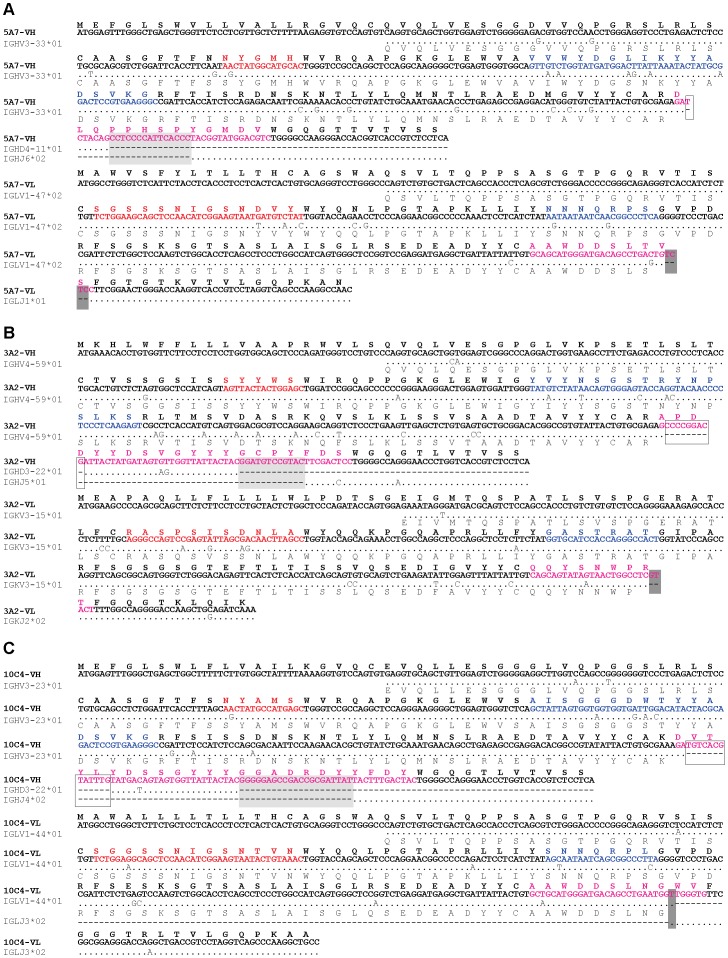
Nucleotide and amino acid sequences of the V_H_ and V_L_ of three HuMAbs 5A7 (A), 3A2 (B) and 10C4 (C). Closest germline sequences in NCBI database by IgBlast softwares were aligned. Complementarity-determining regions (CDRs) are indicated in red, blue and pink (CDRs 1, 2 and 3, respectively). V-D and D-J junctions in VH and V-J junctions in VL are shown in white, light gray and dark gray colors, respectively.

Healthy volunteers were vaccinated with the trivalent HA split vaccine including A/Brisbane/59/2007 (H1N1), A/Uruguay/716/2007 (H3N2), and B/Florida/4/2006 strains. Then, 1–2 weeks later, the vaccine-derived PBMCs were fused with SPYMEG cells. After screening for MAb specificity to influenza viruses, the cells in the specific MAb-positive wells were cloned by limiting dilution. Ultimately, three hybridoma clones producing HuMAbs, designated 5A7, 3A2 and 10C4, were established against influenza B virus. These HuMAbs did not react with influenza A virus. HuMAb reactivity was tested by immunofluorescence assay (IFA) and western blotting using Madin-Darby canine kidney (MDCK) cells infected with B/Florida/4/2006, homologous with the vaccine antigen. All three HuMAbs reacted with HA protein expressed by transfection into 293T cells (Table S1). IgG isotyping was performed by ELISA and revealed that HuMAbs 5A7 and 10C4 were IgG1, and 3A2 was IgG3 (Table S1). Sequences of the V_H_ and V_L_ region of the three HuMAbs were compared and analyzed to the closest germline sequences using IgBlast software in NCBI database. These three HuMAbs were derived from different germ lines except D region in V_H_ of 3A2 and 10C4 (Figure 1).

### Neutralizing activities of HuMAbs

**Figure 2:**
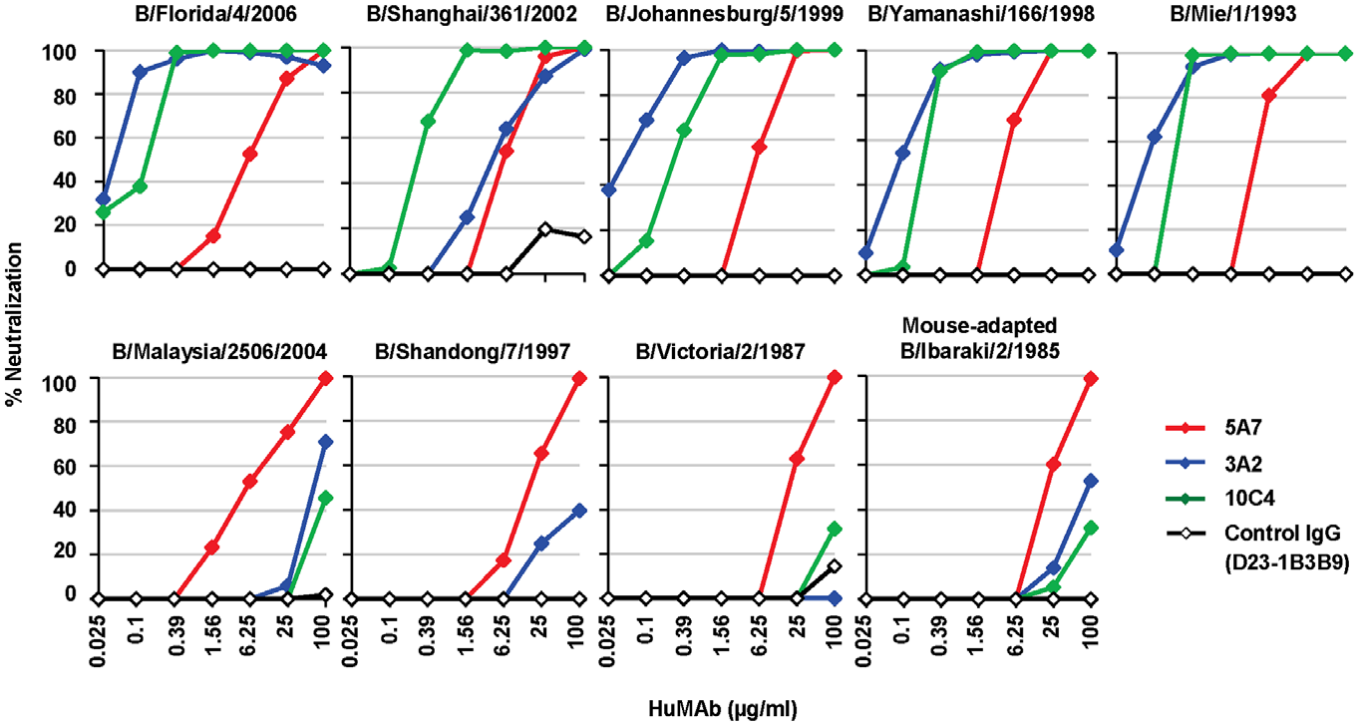
Reactivity of three HuMAbs. *In vitro* VN assay was performed with HuMAbs 5A7 (red), 3A2 (blue),10C4 (green) and control IgG (D23-1B3B9; black). HuMAbs (100 µg/ml) were serially four-fold diluted. The percentage of neutralization was estimated as the viral infectivity under HuMAb-treated conditions compared with that without HuMAb. Upper panels are Yamagata lineage viruses and lower panels are Victoria lineage viruses.

Next, the three HuMAbs were evaluated for their ability to neutralize influenza B viruses by *in vitro* virus neutralization (VN) assay (Figure 2). The VN test was carried out on MDCK cells infected with influenza B virus under the treatment with serial four-fold dilutions of HuMAbs. The viruses used were B/Florida/4/2006, B/Shanghai/361/2002, B/Johannesburg/5/1999, B/Yamanashi/166/1998 and B/Mie/1/1993 for the Yamagata lineage, and B/Malaysia/2506/2004, B/Shandong/7/1997 and B/Victoria/2/1987 for the Victoria lineage. The mouse-adapted B/Ibaraki/2/1985 in the Victoria lineage, used in the passive transfer experiment described below, was also subjected to VN assay. HuMAb 5A7 showed a lower neutralizing activity compared with 3A2 and 10C4 against the Yamagata lineage; however, 5A7 neutralized all strains in the Yamagata and Victoria lineages that were isolated during 1985 to 2006. HuMAbs 3A2 and 10C4 neutralized the Yamagata lineage effectively, whereas they had little neutralization effect on all the Victoria lineage viruses. An anti-dengue virus HuMAb (D23-1B3B9) derived from PBMCs of a patient infected with dengue virus serotype 2 [33] was used as a control IgG. It did not neutralize any influenza B viral strains.

To clarify the mechanism of neutralization by the three HuMAbs, hemagglutinin inhibition (HI) and fusion inhibition assays were performed. All three HuMAbs had HI activity; 3A2 and 10C4 showed markedly higher HI titers (0.39 µg/ml) than 5A7 (25.0 µg/ml). Fusion inhibition assay showed that they all had the ability to inhibit cell-cell fusion; the concentration of HuMAb necessary for complete inhibition was lower for 3A2 and 10C4 (25 µg/ml) than 5A7 (100 µg/ml) at pH 5.5 (Figure S1). By contrast, the control IgG did not show any HI or fusion inhibition activity, even at 100 µg/ml.

In addition, HuMAbs 5A7, 3A2 and 10C4 were subjected to surface plasmon resonance analysis to examine their binding affinities. Each HuMAb was immobilized on the surface of the sensor chip. The vaccine antigen, HA protein of B/Florida/4/2006, at concentrations 12.5, 25, 50, 100 and 200 nM was consecutively injected on the chip surface and the association and dissociation phases were monitored. K_D_ value could not be calculated for 5A7 precisely as it was difficult to dissociate from HA (Table 1 and Figure S2). However, the estimated K_D_ value of 5A7 (less than 5.6×10^−9^ M) was similar to that of 10C4 (1.8×10^−9^ M). By contrast, the control IgG did not associate with HA at all (data not shown).

**Table 1:** Kinetic constants of HuMAbs binding to influenza B virus-derived HA.

	*k*on^1^ (S^−1^)	*k*off^2^ (M^−1^S^−1^)	K_D_ (M)
5A7	1.8×10^3^	<1.0×10^−5^	<5.6×10^−9^
3A2	5.3×10^4^	2.1×10^−5^	4.0×10^−10^
10C4	1.6×10^4^	2.8×10^−5^	1.8×10^−9^

1 Association rate constant.

2 Dissociation rate constant.

### Epitope mapping of three HuMAbs

**Figure 3:**
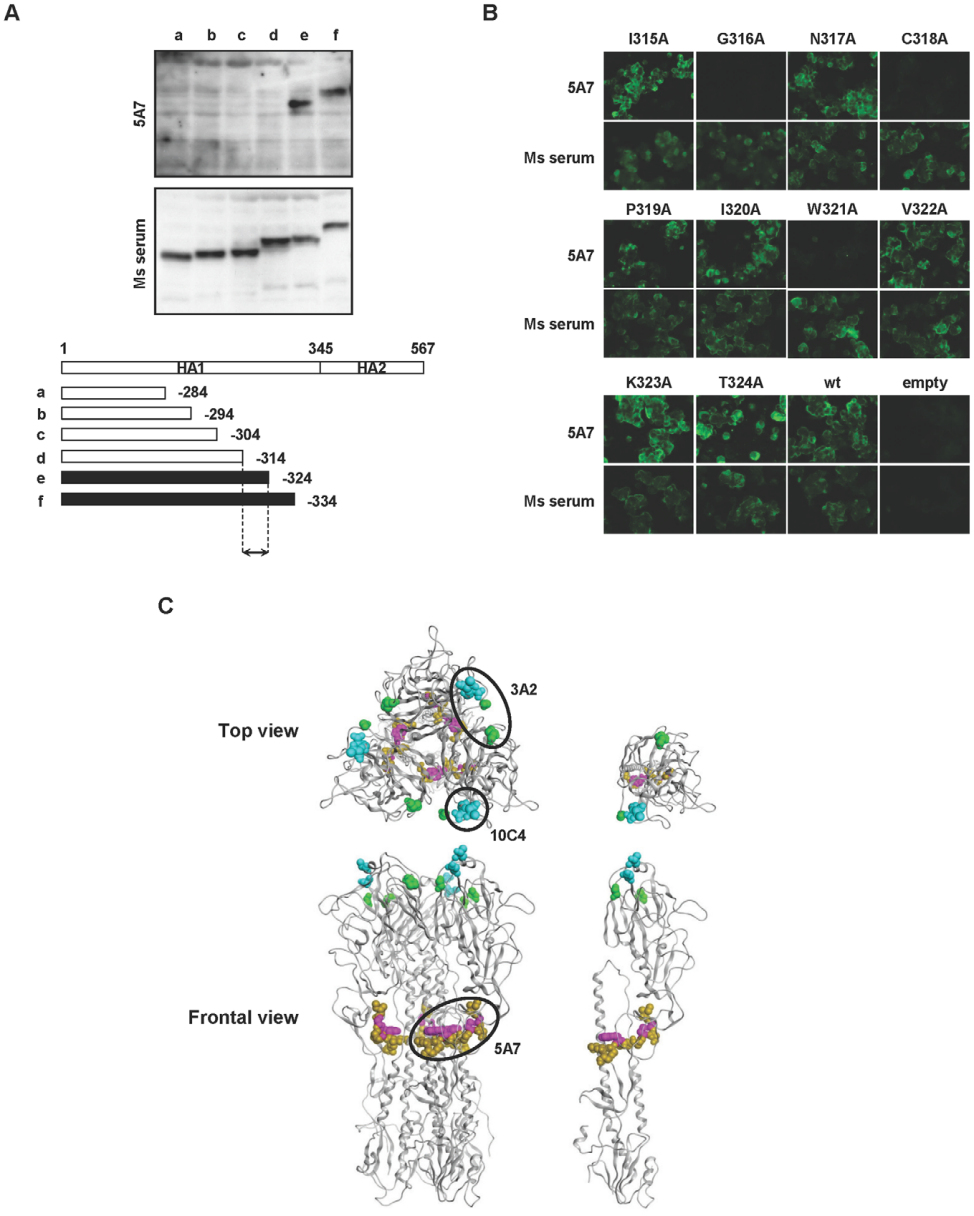
Epitope mapping of HuMAbs. (A) Reactivity of 5A7 with truncated HA. A series of truncated forms of HA (a to f) were prepared in an expression plasmid as depicted in the diagram. Transfected 293T cells were subjected to western blotting with 5A7 or serum from a mouse infected with B/Ibaraki/2/1985 as a control (Ms serum). Black bars in the HA diagram indicate reactivity with 5A7. Arrowed region is the estimated region involved in recognition. (B) Expression plasmids bearing one point mutated B/Florida/4/2006 HA protein were prepared. 293T cells expressing the mutated HA were subjected to IFA with 5A7 (upper panels) or Ms serum as a control (lower panels). Wild type B/Florida/4/2006 (wt) and pCAGGSII vector without an insertion (empty) were used as controls. (C) Epitope map of the HuMAbs in the three-dimensional structure of the HA trimer (left models) and monomer (right models). The amino acid positions identified by epitope mapping are 315 to 324 (dark yellow), 316, 318 and 321 (pink), 194 and 196 (blue), 131 and 227 (green). The epitope region of each HuMAb is circled.

Next, the epitope regions recognized by the three HuMAbs were determined. At first, escape mutants were selected by culturing B/Florida/4/2006 in the presence of serial ten-fold diluted HuMAb. MDCK cells were infected with the mixture in a 24-well plate and 72 hours later the supernatants were subjected to VN and HI assays, and direct sequencing analysis of the HA gene (Figure S3). Escape mutants were not established for 5A7, even after the virus was serially passaged 10 times in the presence of this HuMAb. 5A7 reacted with the HA protein by western blotting under reducing conditions. Therefore, the region of 5A7 involved in recognition was refined using HA truncation vectors containing HA segments of varying length (Figure 3A). Western blotting with 5A7 was carried out on 293T cells transfected with the truncated HA expression vectors. HuMAb 5A7 reacted with truncated HA segments that included amino acid residues 1–324 but not with those with residues 1–314 (Figure 3A): amino acid numbering was started after the signal peptide [34]. These results indicate that 5A7 recognizes amino acid residues between 315 to 324 (IGNCPIWVKT) in the HA protein, which locates near the C terminal of the HA1 protein. The epitope region of 5A7 was determined by mutating each of the 315–324 amino acid residues singly. Each residue was replaced by alanine using a site-directed mutagenesis method. Mutant HAs expressed in 293T cells were tested for reactivity with 5A7 by IFA. Mutants G316A, C318A and W321A did not react with 5A7 (Figure 3B), indicating that 316G, 318C and 321W amino acids critically affected the structure of the epitope of 5A7. To estimate the conservation of the amino acid sequences in the epitope of 5A7, HA sequences of influenza B virus were extracted from the NCBI database. Notably, 2,851 among 2,853 viral sequences (99.93%) showed an identical amino acid sequence in the epitope region (Table S2). Among the Yamagata and Victoria lineages, there was only 1 divergent strain, respectively.

By contrast, escape mutants were obtained in the presence of 3A2 and 10C4. They showed four-fold reduced VN and HI activities compared with the parent virus after just one passage of the virus (Figure S3). Each escape mutant obtained in the presence of 3A2 and 10C4 had amino acid substitutions at identical positions, 194D and 196T (Figure S4), both located in the readily mutable 190-helix antigenic site near the receptor binding site [34]. In addition, the different amino acid residues in V_H_ and V_L_ of 3A2 and 10C4 (Figure 1) indicated that these two HuMAbs were derived from different germ lines. These results are consistent with 3A2 and 10C4 reactivity with only the Yamagata strains, since Yamagata and Victoria strains differ in amino acid sequence at this position. HuMAb 3A2 showed low reactivity against B/Shanghai/361/2002 (Figure 2A) and was therefore examined for an additional distinct epitope region. To do this, various chimeric sequences of HA were constructed from B/Florida/4/2006 and B/Shanghai/361/2002, which differ at seven residues (positions 37, 40, 88, 131, 227, 249 and 456), expressed in plasmids, and transfected into 293T cells. IFA of the chimeric HA proteins expressed in 293T cells showed that 131P and 227S were essential for reaction with 3A2 (Figure S5). These results indicate that the epitope of 3A2 is dependent on residues at positions 131, 194, 196 and 227, and the epitope of 10C4 is dependent on residues at positions 194 and 196.

The epitope regions to which the three HuMAbs map are shown in an HA monomer and trimer three-dimensional models in Figure 3C. HuMAbs 3A2 and 10C4 recognized the top of the globular head including the 190-helix antigenic site, whereas 5A7 reacted with the stalk region distant from the viral membrane.

### Therapeutic efficacy of 5A7 *in vivo*

**Figure 4:**
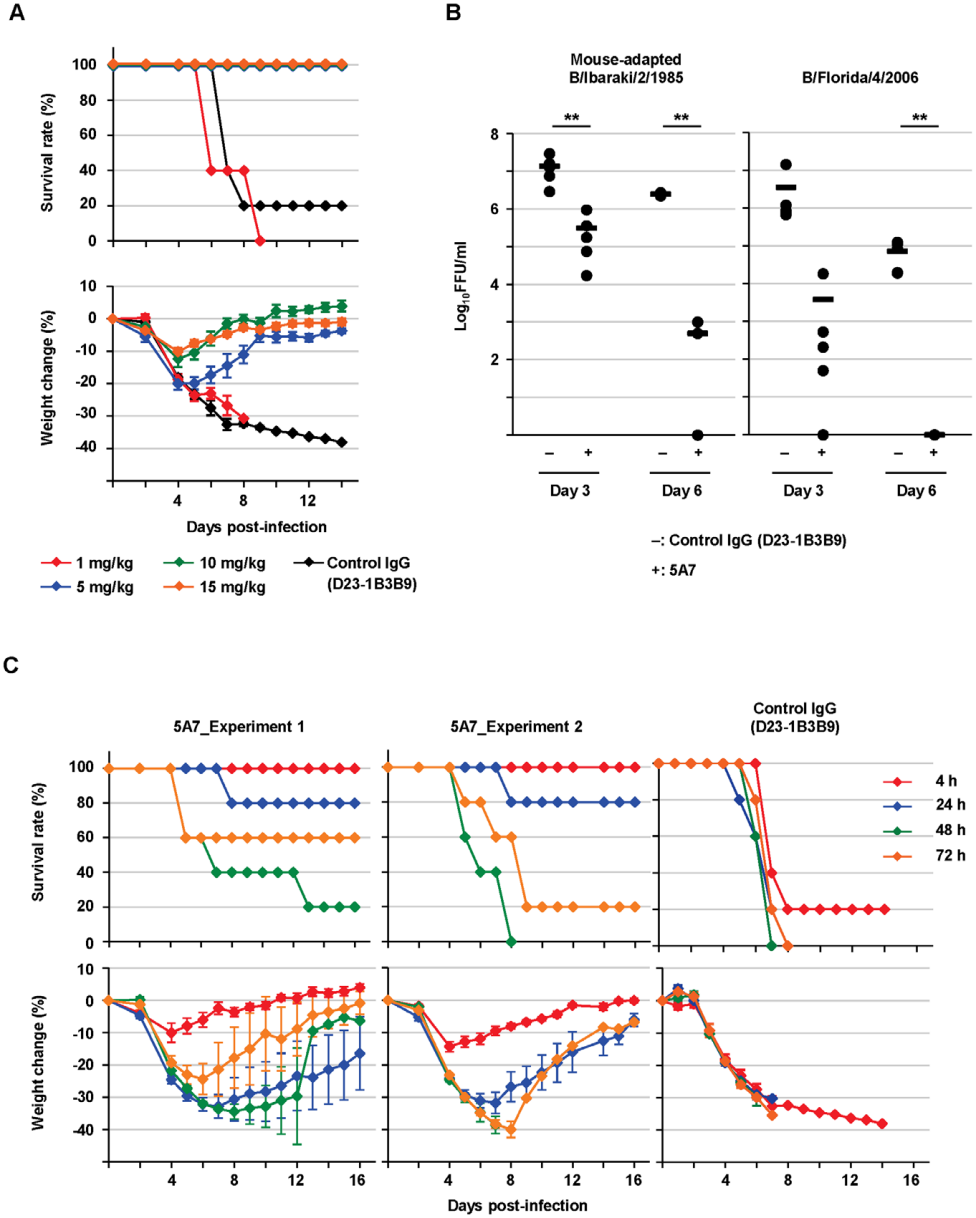
Therapeutic efficacy of 5A7 in mice. (A) Mice were treated intraperitoneally with HuMAb 5A7 at 1 (red line), 5 (blue), 10 (green), or 15 (orange) mg/kg or with control IgG at 10 mg/kg (black) 4 hours after intranasal injection of a lethal dose (1.47×10^3^ MLD_50_/mouse) of mouse-adapted B/Ibaraki/2/1985. Survival and body weight were checked daily. Each group consists of five mice. Body weight is shown as the mean ± SEM of five mice. (B) Mice were treated with 5A7 (+) or control IgG (D23-1B3B9; −) at 10 mg/kg 4 hours post-infection with 1.47×10^3^ MLD_50_/mouse of mouse-adapted B/Ibaraki/2/1985 (left panel) or 5.0×10^3^ FFU/mouse of B/Florida/4/2006 passaged eight times in mouse lung (right panel). The titers in lungs were calculated at day 3 and day 6 post-infection. Each group consists of five mice (except control IgG-treated group with mouse-adapted B/Ibaraki/2/1985 at day 6, which consists of four mice as one accidentally died before the lung could be collected). Black bars are mean values. **: *P*<0.01 compared to control IgG-treated group. (C) Two independent experiments were similarly performed (Left two panels). Mice were given 10 mg/kg HuMAb 5A7 at 4 (red line), 24 (blue), 48 (green) or 72 (orange) hours post-infection with mouse-adapted B/Ibaraki/2/1985 (1.47×10^3^ MLD_50_/mouse). Right panel shows 10 mg/kg control IgG-treated group at 4, 24, 48 or 72 hours post-infection. Survival and body weight were checked daily. Each group consists of five mice per experiment. Body weight is shown as the mean ± SEM of five mice.

The evaluation of 5A7 as a passive transfer therapy for influenza B viral infection was examined in mice. Six-week-old mice were treated intraperitoneally with 5A7 at 1, 5, 10 or 15 mg/kg or with control IgG at 10 mg/kg, 4 hours after an intranasal injection with a lethal dose (1.47×10^3^ 50% mouse lethal dose (MLD_50_)/mouse) of mouse-adapted B/Ibaraki/2/1985. Survival rate and body weight change were checked daily. When body weight decreased to less than 60% of starting weight, mice were sacrificed. With respect to survival rate, complete therapeutic efficacy against the virus challenge was seen with 5, 10 and 15 mg/kg of 5A7 examined (Figure 4A, Upper panel). The weight change was mild in the groups, especially in those treated with 5A7 at 10 or 15 mg/kg (Figure 4A, lower panel).

Next, viral load in the lungs was titrated. Mice infected with mouse-adapted B/Ibaraki/2/1985 (1.47×10^3^ MLD_50_/mouse), or B/Florida/4/2006 that had been passaged eight times in mice lungs (5.0×10^3^ focus-forming units (FFU)/mouse), were treated with 5A7 or control IgG at 10 mg/kg 4 hours post-infection, and then sacrificed on day 3 and day 6 post-infection. The lungs were homogenized and tested in focus-forming assays with MDCK cells. The viral titers were significantly lower in 5A7-treated mice compared to the control IgG-treated group for both viral infections (Figure 4B). Finally, mice were treated with 5A7 or control IgG at 10 mg/kg intraperitoneally at 4, 24, 48 or 72 hours after an intranasal injection with a lethal dose (1.47×10^3^ MLD_50_/mouse) of mouse-adapted B/Ibaraki/2/1985. Survival rate and body weight change were monitored (Figure 4C). Two independent experiments were similarly performed for 5A7 treatment with five mice/group/experiment (Experiments 1 and 2 in Figure 4C). When administered 4 hours post-infection, 5A7 treatment showed complete therapeutic efficacy, as was observed above (shown in Figure 4A). Notably, 80% of mice were alive in the group treated with 5A7 24 hours post-infection. Moreover, several mice survived after treatment with 5A7 48 hours post-infection and, surprisingly, more mice survived when treated 72 hours post-infection. By contrast, 80% of mice treated with control IgG at 4 hours post-infection died and the surviving 20% did not show recovery of body weight during the experiment. All of mice died by 8 days post-infection in the groups treated with control IgG after 24 hours post-infection (control IgG in Figure 4C).

## Discussion

A broadly neutralizing HuMAb, 5A7, was established using PBMC from a vaccinated healthy volunteer. This antibody neutralizes influenza B virus strains isolated between 1985 and 2006 that belong to the Yamagata or Victoria lineage. Moreover, therapeutic efficacy was shown in mice even when the HuMAb was administered 48 or 72 hours after viral challenge. As previously reported for almost all MAbs broadly neutralizing influenza virus, 5A7 recognized the stalk region of the HA protein [26]–[29]. Importantly, the epitope region recognized by 5A7 is highly conserved in influenza B virus and the divergent strains occur only sparsely (Table S2). Although influenza B virus has a much slower mutational rate than that observed for influenza A subtypes like H1 and H3, cocirculation of two phylogenetically and antigenically distinct lineages of influenza B virus leads to annual epidemics in humans [6], [7]. Dreyfus *et al.* first reported broadly neutralizing HuMAbs against influenza B virus in September 2012 [30]. The epitope regions they identified were distinct from that of our HuMAb, 5A7. Characterization of the epitope region recognized by such a HuMAb could therefore provide insight for the development of a universal vaccine.

The high degree of conservation of amino acid residues in the epitope region implies that influenza B virus would not easily induce mutation in this region. Indeed, amino acid residues in the epitope region did not mutate even when the virus was passaged ten times under 5A7-treatment conditions. It could be considered that the poor inhibitory activity of 5A7 (weak HI, fusion inhibition and VN_50_) is the cause of failure to establish escape mutants. However, a previous report showed that escape mutants could be prepared even from an MAb with weak fusion inhibition and complete neutralizing activities (25 and 50 µg/ml, respectively), and without HI activity, in order to map its epitope [35], [36]. Since 5A7 shows similarly weak activity, we would also expect to have been able to prepare 5A7 escape mutants and determine the epitope region. Failure to establish escape mutants in the presence of 5A7 could be an advantage for its development as a therapeutic tool, and also for designing the next generation of globally effective vaccines.

Generally, MAbs recognizing the globular head show strong HI activity, whereas those against the stalk region usually show none [35]. Thus, it is considered that MAbs against the globular head inhibit the receptor binding step, while MAbs against the stalk region inhibit the fusion step, in viral replication [26], [37]. In fact, HuMAbs, 3A2 and 10C4, that recognize the 190-helix in the globular head near the receptor binding site, showed strong HI activity, which suggests that they can inhibit viral binding to the receptor on the host cells. Their ability to inhibit the fusion process (Figure S1) implies that they could secondarily disturb the low pH-dependent structural change in HA by binding to the globular head. Surprisingly, although 5A7 reacted to the stalk region it also showed specific and weak HI activity. This suggests that MAb recognizing the stalk region distal from the viral membrane could affect the ability of the virus to bind to the receptor. As reported for MAbs reacting to the stalk region, 5A7 also showed specific fusion inhibition activity [28], [38]. These results suggest that HuMAb 5A7 could inhibit viral entry by preventing receptor binding, and the subsequent fusion process.

Previous reports show that the concentration of MAbs necessary for viral neutralization was much higher for those recognizing the stalk region of the envelope protein than for those reacting with the globular head in influenza viruses [28], [36], [39], as well as other viruses [40]. In agreement with these reports, the concentration required for viral neutralization in this study was higher for 5A7 than 3A2 and 10C4 (Figure 2). Such results can be explained by either a difference in binding affinity or in physical accessibility of HuMAbs to the epitope region. 5A7 showed similar K_D_ with 10C4 in binding kinetics analysis (Table 1 and Figure S2), indicating that 5A7 has more difficulty physically accessing the epitope region of the HA protein. Modifying the HuMAb structure to enable easier access to the epitope region and improving its binding affinity, as described [41], [42], could lead to the development of better therapeutic HuMAbs for influenza.

HuMAb 5A7 had specific therapeutic efficacy in mice even when administered after viral challenge (Figure 4C). Two independent passive transfer experiments were performed. Surprisingly, in both experiments, mice treated with 5A7 HuMAb 72 hours post-infection had a better survival rate than those treated 48 hours post-infection. It is reported that in the first 24 hours after infection, the levels of lymphocyte apoptosis increase transiently in both nasal-associated lymphoid tissue and spleen, and cellular immune suppression occurs [43]. Temporal cellular immune suppression or infection-mediated endogenous signals could therefore interfere with the efficacy of exogenous HuMAb. These results imply that the timing of HuMAb treatment could be critical for efficacy, and therefore, injection at several time points may be necessary.

Mouse-adapted B/Ibaraki/2/1985 was used to examine the kinetics of survival rate and body weight change in passive transfer experiments because other viral strains are not lethal to mice, even if passaged several times *in vivo*. HuMAb 5A7 protected mice against mouse-adapted B/Ibaraki/2/1985 viral challenge although 5A7 was obtained from a volunteer vaccinated with B/Florida/4/2006 and had shown the lowest sensitivity to this viral strain *in vitro* (Figure 2). These results suggested that 5A7 would have therapeutic efficacy against a wide spectrum of influenza B viruses and, in fact, lung viral titers of both mouse-adapted B/Ibaraki/2/1985 and B/Florida/4/2006 were reduced significantly under 5A7-treatment conditions (Figure 4B). Further study using both mice and ferrets with several viral strains is needed to confirm the wide-ranging therapeutic potential of 5A7 *in vivo*.

## Materials and Methods

### Ethics statement

Human materials were collected using protocols approved by the Institutional Review Boards of the Research Institute for Microbial Diseases, Osaka University (#19-8-6). Written informed consent was obtained from the participants. Animal studies were conducted under the applicable laws and guidelines for the care and use of laboratory animals in the Research Institute for Microbial Diseases, Osaka University. They were approved by the Animal Experiment Committee of the Research Institute for Microbial Diseases, Osaka University (#H21-24-0), as specified in the Fundamental Guidelines for the Proper Conduct of Animal Experiment and Related Activities in Academic Research Institutions under the jurisdiction of the Ministry of Education, Culture, Sports, Science and Technology, Japan, 2006.

### HuMAb preparation

HuMAbs were prepared as described previously [31]. Briefly, 10 ml blood was drawn from a healthy volunteer vaccinated in the 2008/2009 winter season with trivalent HA split vaccine, which included A/Brisbane/59/2007, A/Uruguay/716/2007, and B/Florida/4/2006 (The Research Foundation for Microbial Diseases of Osaka University, Osaka, Japan), and PBMCs were collected by density gradient centrifugation through Ficoll-Paque Plus (GE Healthcare). SPYMEG cells were used as fusion partner cells. SPYMEG cells, which are non-secretors of human and murine immunoglobulins, were established by fusion between mouse myeloma cell line SP2/0-Ag14 and human megakaryoblastic cell line MEG-01 [31]. The PBMCs were fused with SPYMEG cells using polyethylene glycol #1500 (Roche). The fused cells were cultured in Dulbecco's modified Eagle medium (DMEM; Invitrogen) supplemented with 15% fetal bovine serum and hypoxanthine-aminopterin-thymidine. The first screening for antibody specificity to influenza virus was performed by IFA. Cells in the specific MAb-positive wells were then cloned by limiting dilution, followed by a second screening by IFA. Hybridoma cells taken from IFA-positive wells that had a single colony per well were cultured and expanded in Hybridoma-SFM (Invitrogen). MAb was purified from 100 ml hybridoma culture supernatant by affinity chromatography using HiTrap Protein G HP Columns (GE Healthcare) and then dialyzed into phosphate buffered saline (PBS) using Slide-A-Lyzer Dialysis Cassettes (Thermo Scientific). Control IgG (D23-1B3B9) used in this study was derived from the PBMCs of a dengue patient infected with dengue virus serotype 2 [33].

### Viruses

Eight influenza B vaccine strains (B/Florida/4/2006, B/Malaysia/2506/2004, B/Shanghai/361/2002, B/Johannesburg/5/1999, B/Yamanashi/166/1998, B/Shandong/7/1997, B/Mie/1/1993, and B/Victoria/2/1987) and a mouse-adapted influenza B virus strain (B/Ibaraki/2/1985) were used. The B/Malaysia/2506/2004 and B/Florida/4/2006 strains were kindly provided by the National Institute of Infectious Diseases, Tokyo, Japan. The mouse-adapted B/Ibaraki/2/1985 strain was provided by Dr. S. Tamura, National Institute of Infectious Diseases [44]. Viruses were propagated either in MDCK cells or in 9-day-old embryonated chicken eggs. Infectivity was titrated by focus-forming assay: MDCK cells in a 96-well plate were adsorbed with viruses serially ten-fold diluted at 37°C for 1 hour. The cells were then washed with PBS and incubated at 37°C for 12 hours. Cells were fixed and subjected to IFA.

### Characterization of HuMAbs

For IFA, the infected cells were fixed with absolute ethanol for 2 minutes at room temperature and then reacted with hybridoma supernatant for 30 minutes at 37°C, followed by incubation with FITC-conjugated anti-human IgG for 45 minutes at 37°C. For western blotting, the infected samples in a loading buffer containing 2-mercaptoethanol were used for electrophoresis and then blotted to PVDF membrane. They were probed with hybridoma supernatant for 1 hour at 37°C, followed by incubation with HRP-conjugated anti-human IgG for 1 hour at 37°C.

### VN assay

The VN test was carried out as described previously [45], with minor modifications. HuMAb at a concentration of 100 µg/ml was serially four-fold diluted with Minimum Essential Medium (MEM; Invitrogen) and incubated with 200 FFU of viruses at 37°C for 1 hour. Then, MDCK cells were adsorbed with the mixtures at 37°C for 1 hour. After incubation for 12 hours, the cells were fixed and subjected to IFA.

### HI assay

First, viral titers were determined with a hemagglutination assay. Briefly, the viruses were serially diluted two-fold with PBS and mixed with 0.7% (v/v) human O-type red blood cells. After incubation at room temperature for 1 hour, hemagglutination units (HAUs) were estimated. Next, HI titration was performed as follows: MAb at a concentration of 100 µg/ml was serially two-fold diluted and mixed with 8 HAU per 50 µl of viral sample. After incubation at 37°C for 1 hour, the mixtures were further incubated with 0.7% (v/v) human red blood cells for 1 hour at room temperature. The lowest concentration of HuMAb that completely inhibited hemagglutination was designated the HI titer.

### Fusion inhibition assay

Cell-cell fusion was accomplished as described previously [35]. Briefly, monkey kidney cell line CV-1 cells were infected with B/Florida/4/2006 at an MOI of 0.3. After incubation for 24 hours, the cells were washed with MEM and then incubated for 15 minutes at 37°C in MEM supplemented with 2.5 µg/ml of acetylated trypsin (Sigma). After washing, the cells were incubated for 30 minutes with diluted HuMAbs. Thereafter, the cells were treated for 2 minutes at 37°C with MEM supplemented with 10 mM MES and 10 mM HEPES (pH 5.5). After the medium was completely removed by washing, the cells were incubated for 3 hours. Then they were fixed with absolute methanol and stained with Giemsa (Wako).

### Surface plasmon resonance analysis (binding kinetics analysis)

Surface plasmon resonance analysis was performed [46] with a Biacore T200 (GE Healthcare). Interaction was measured in running buffer (10 mM HEPES-Na pH 7.5, 150 mM NaCl, 3 mM EDTA, 0.005% Tween 20) at 25°C. The surface of a CM5 sensor chip was coated with goat anti-human IgG Fcγ antibody (Jackson ImmunoResearch) at a density of 10,000 resonance units (RU) by an amine coupling technique using 1-ethyl-3-[3-dimethylaminopropyl] carbodiimide hydrochloride and Sulfo-NHS for activation, and ethanolamine for blocking. As the ligand, each of the anti-HA HuMAbs was captured on the chip surface via anti-human IgG Fcγ to a density of 10–80 RU. The HA protein of B/Florida/4/2006 vaccine antigen (The Research Foundation for Microbial Diseases of Osaka University) was diluted in running buffer at concentrations of 12.5, 25, 50, 100 and 200 nM (as monomers). Each concentration was injected sequentially on to the chip at a flow rate of 60 µl/min for a contact time of 60 seconds, and then washed with the running buffer for a dissociation time of 30 minutes. Regeneration of the chip surface was performed with 10 mM glycine-HCl pH 1.5. Binding kinetics were evaluated using Biacore T200 evaluation software version 1.0 (GE healthcare) using the single kinetics analysis method with a 1∶1 binding model.

### Selection of escape mutants

Escape mutants were selected by culturing B/Florida/4/2006 in the presence of HuMAb as described previously [47], with minor modifications. Viruses (to give final concentrations of 100 to 1,000 FFU/ml) were incubated with HuMAb serially ten-fold diluted (to give final concentrations of 0.0025, 0.025, 0.25 and 2.5 µg/ml), at 37°C for 1 hour. Then, MDCK cells in a 24-well plate were inoculated with the mixtures (n = 6 for each) and cultured in DMEM/F-12+GlutaMAX-I supplemented with 0.4% bovine serum albumin (BSA), antibiotics and 2 µg/ml acetylated trypsin. After culturing for 72 hours, the supernatants in each well were collected separately and subjected to VN and HI assays. The entire HA gene was directly sequenced from the mixed population in the supernatants of those viral samples that showed a reduced neutralization and HI titer of more than four-fold.

### Direct sequencing analysis

Viral RNA extracted with QIAamp Viral RNA Mini Kit (Qiagen) was subjected to one step RT-PCR (Superscript III One-Step RT-PCR System with Platinum *Taq* High Fidelity; Invitrogen) with the following HA primer set: 5′-CAGAATTCATGAAGGCAATAATTGTACTAC-3′ forward and 5′-CTCfCGCGGCCGCTTATAGACAGATGGAGCATGAAACG-3′ reverse. The PCR products were purified with Qiaquick PCR Purification Kit (Qiagen). After electrophoresis, the discrete band was extracted using the Qiaquick Gel Extraction Kit (Qiagen) and sequenced.

### Construction of HA plasmids

HA gene of B/Florida/4/2006 and B/Shanghai/361/2002 was amplified by one step RT-PCR, as described above, and inserted into the pGEM-T Easy Vector (Promega). Mutant and truncated HA genes were generated by site-directed mutagenic PCR (GeneTailor Site-Directed Mutagenesis System; Invitrogen) and conventional PCR (Expand High Fidelity^PLUS^ PCR System; Roche), respectively, using the HA plasmid inserted into pGEM-T easy vector. Each of the plasmids was subcloned into the expression vector pCAGGS/MCSII [48]. The expression plasmids were transfected into human embryonic kidney 293T cells with lipofectamine 2000 (Invitrogen) according to the manufacturer's instructions.

### Homology of amino acid sequence

Amino acid sequence and sequence information were downloaded from the FTP site of Influenza Virus Resource (ftp://ftp.ncbi.nih.gov/genomes.INFLUENZA/) on January 31, 2012. HA sequences of influenza B virus were extracted from the database, and then aligned using the MAFFT program [49], [50]. The number of variants in the target sequence (IGNCPIWVKT) was counted.

### IgG isotyping

ELISA microplates (MaxiSorp; Nunc) were coated overnight at 4°C with goat anti-human IgG (Jackson ImmunoResearch Laboratories) in 0.05 M sodium bicarbonate buffer (pH 8.6). After washing with PBS including 0.1% Tween-20, the wells were blocked with 0.5% BSA in PBS for 1 hour at 37°C. After washing again, the wells were incubated with hybridoma supernatants or control serum for 2 hours at 37°C. Following further washing, the wells were incubated with HRP-conjugated anti-human IgG1, IgG2, IgG3 or IgG4 (SouthernBiotech) for 1 hour at 37°C. The wells were washed five times followed by incubation with TMB peroxidase substrate (KPL) at room temperature in the dark. After 20 minutes, the reaction was stopped with 2N H_2_SO_4_ solution. The color development was read at 450 nm in an ELISA Photometer (Biotek ELISA Reader; Biotek). All samples were run in triplicate.

### Sequencing of HuMAb variable regions

Total RNA extracted from the hybridoma using an RNeasy Mini Kit (Qiagen) was subjected to RT-PCR using a PrimeScript RT reagent Kit (Takara) with an oligo (dT) primer. The coding region of the H- and L-chains of HuMAb was amplified by PCR with the following primers: 5′-ATGGAGTTTGGGCTGAGCTGGGTT-3′ (H-chain-forward) and 5′-CTCCCGCGGCTTTGTCTTGGCATTA-3′ (H-chain-reverse); and 5′-ATGGCCTGGRYCYCMYTCYWCCTM-3′ (L-chain-forward) and 5′-TGGCAGCTGTAGCTTCTGTGGGACT-3′ (L-chain-reverse). PCR products were ligated into pGEM-T Easy Vector (Promega) and their sequences were analyzed using a BigDye Terminator v3.1 Cycle Sequencing Kit and an ABI Prism 3100 Genetic Analyzer (Applied Biosystems). Determined sequences were analyzed and compared to the NCBI database using the IgBlast softwares (http://www.ncbi.nlm.nih.gov/igblast/).

### Molecular modeling

The HA structure was constructed using Molecular Operating Environment software (Chemical Computing Group Inc.) based on the crystal structure of B/Hong Kong/8/1973 (PDB ID: 2RFU) [51].

### Passive transfer experiments

All mice used were 6-week-old female BALB/c mice from Japan SLC Inc. Before infection, mice were anesthetized by intraperitoneal administration of pentobarbital sodium (Somnopentyl; Kyoritsu Seiyaku Corporation). MLD_50_ was determined by inoculating intranasally with serial 10-fold dilutions of virus and calculating with the Reed-Muench method [52]. For passive transfer experiments, mice were treated with HuMAb at 1, 5, 10 or 15 mg/kg intraperitoneally at 4, 24, 48 or 72 hours post-infection with 25 µl mouse-adapted B/Ibaraki/2/1985 virus at a lethal dose (1.47×10^3^ MLD_50_/mouse). Mice were weighed daily and sacrificed if they fell to 60% of starting weight. To titrate the viruses in the infected lungs, mouse-adapted B/Ibaraki/2/1985 or B/Florida/4/2006 passaged eight times in mice lungs were infected at 1.47×10^3^ MLD_50_/mouse or 5.0×10^3^ FFU/mouse, respectively. The lungs were harvested on day 3 and day 6 post-infection and virus titers in lung homogenates were determined by focus-forming assay.

### Statistical analyses

Data are expressed as the means ± standard errors of the means (SEM). Statistical analysis was performed by Student's *t* test. A *P* value of <0.05 was considered significant.

### Accession numbers

The GenBank (http://www.ncbi.nlm.nih.gov/GenBank/) accession numbers for the genes discussed in this paper are as follows: IgG-V_H_ in clone 5A7 (AB729122), IgG-V_L_ in clone 5A7 (AB729123), IgG-V_H_ in clone 3A2 (AB729120), IgG-V_L_ in clone 3A2 (AB729121), IgG-V_H_ in clone 10C4 (AB729124), and IgG-V_L_ in clone 10C4 (AB729125).

## Supporting Information

Figure S1 **Cell-cell fusion inhibition assay.** CV-1 cells were infected with B/Florida/4/2006 treated with HuMAbs 5A7, 3A2, 10C4 or control IgG at pH 5.5. HuMAbs (100 µg/ml) were serially four-fold diluted. As a control for pH, assay with 5A7 was also performed at pH 7.0 (second row). Mock/– (bottom left panel) is a mock-infected sample without HuMAb at pH 5.5. Infection/– (bottom right panel) is an infected sample without HuMAb at pH 5.5, as a positive control. Arrowheads show major cell-cell fusion bodies.
(PDF)

Figure S2 **Surface plasmon resonance analysis of binding affinity between HuMAbs and the HA protein of B/Florida/4/2006.** HuMAbs 5A7, 3A2 or 10C4 were immobilized on the surface of a CM5 chip via pre-crosslinked anti-human IgG Fcγ antibody. The HA protein at concentrations of 12.5, 25, 50, 100 and 200 nM were consecutively injected onto the chip surface and the association and dissociation phases were monitored. Signal from the chip surface without anti-HA antibody (the reference value) was subtracted from each reading.
(PDF)

Figure S3 **Diagram showing how the escape mutants were obtained.** Four HuMAb concentrations prepared by serial ten-fold dilutions (right lower diagram) were mixed with B/Florida/4/2006 for 1 hour. Then, each mixture was used to infect MDCK cells in six wells (i.e., four groups of six wells) and incubated for 72 hours (for details, see Materials and methods). Groups were graded according to cytopathic effects: all six wells showing no cytopathic effects (white), some wells showing cytopathic effects (gray), and all wells showing cytopathic effects (black). Supernatants from wells colored gray were collected separately and measured for VN and HI activities. When a four-fold reduction in VN and HI assays was not shown by any of supernatants, one sample was mixed with HuMAbs serially 10-fold diluted described above and infected to newly prepared MDCK cells. P1 to P10 indicates passage number. Out of 12 gray wells, two wells for 3A2 and one well for 10C4 (colored red) showed a four-fold reduction in VN and HI activities compared with the parent virus. Gray wells at P10 of 5A7 and gray and red wells at P1 of 3A2 and 10C4 were subjected to direct sequencing analysis of the HA gene.
(PDF)

Figure S4 **The epitope region of 3A2 and 10C4.** Escape mutants were selected by incubation of B/Florida/4/2006 with HuMAbs. Amino acid sequences of the HA protein in the escape mutants were compared with the original B/Florida/4/2006. Asterisks indicate amino acid residues that differed between the original virus and the escape mutants.
(PDF)

Figure S5 **The additional epitope region of 3A2.** Expression plasmids bearing chimeric HA protein were prepared from B/Shanghai/361/2002 (Sh/02) and B/Florida/4/2006 (Flo/06). 293T cells expressing the chimeric protein were subjected to IFA with 3A2 (left panels). White bars represent the amino acid sequence in Sh/02, and black bars represent the amino acid sequence in Flo/06. The different amino acid residues in the HA protein from each of the two viral strains are shown in the top and bottom bars.
(PDF)

Table S1 **Pattern of reactivity of HuMAbs.**
(PDF)

Table S2 **Homology of the epitope region of HuMAb 5A7 among the corresponding sequences derived from NCBI database.**
(PDF)
