# Supplementary figures and images for: Human Monoclonal Antibodies Broadly Neutralizing against Influenza B Virus
Source: PLoS Pathog. 2013 Feb 7;9(2):e1003150. doi: 10.1371/journal.ppat.1003150 (PMC3567173; doi:10.1371/journal.ppat.1003150)

Figure S1

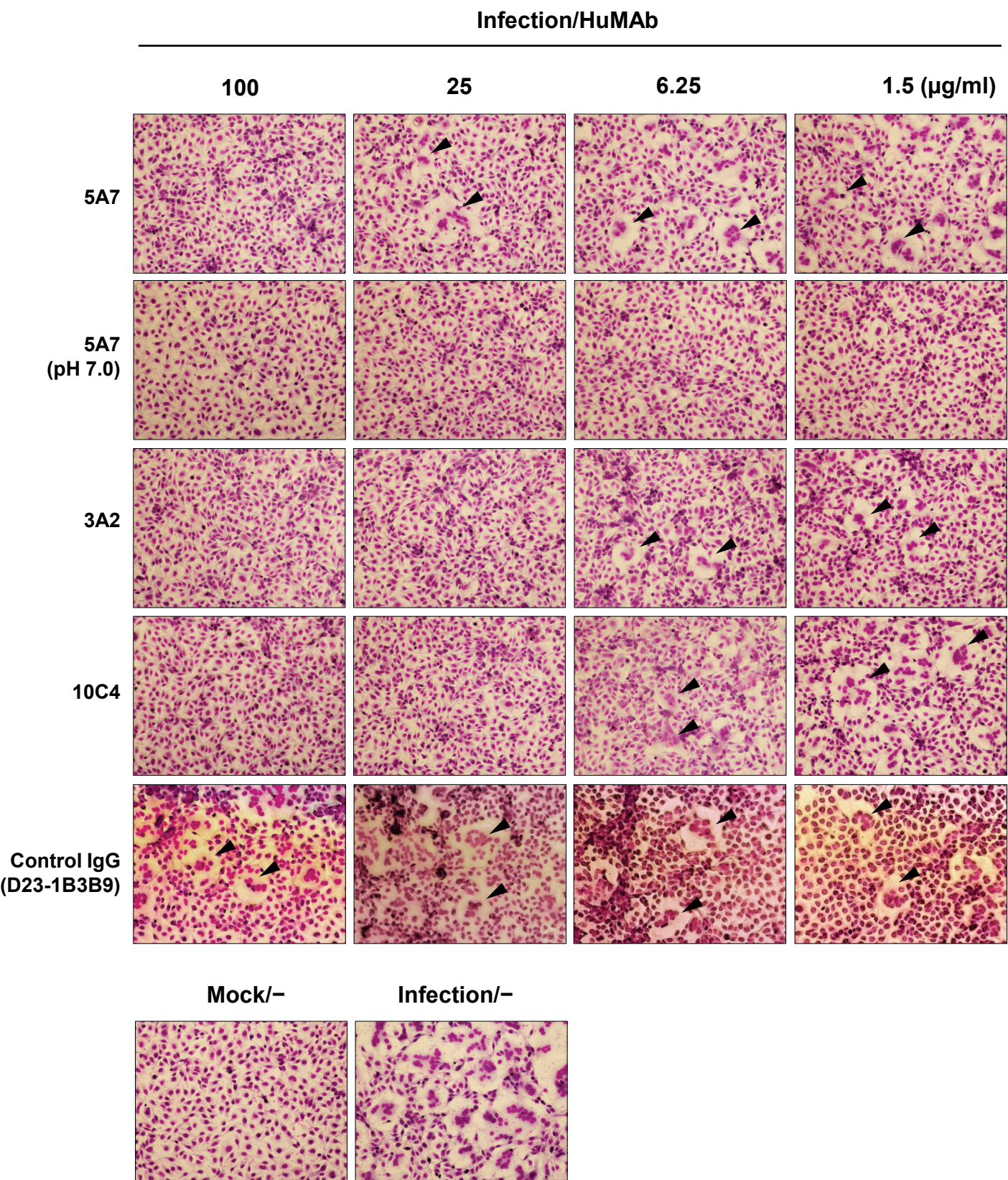

Supplement: Figure S1 — Cell-cell fusion inhibition assay. CV-1 cells were infected with B/Florida/4/2006 treated with HuMAbs 5A7, 3A2, 10C4 or control IgG at pH 5.5. HuMAbs (100 µg/ml) were serially four-fold diluted. As a control for pH, assay with 5A7 was also performed at pH 7.0 (second row). Mock/– (bottom left panel) is a mock-infected sample without HuMAb at pH 5.5. Infection/– (bottom right panel) is an infected sample without HuMAb at pH 5.5, as a positive control. Arrowheads show major cell-cell fusion bodies. (PDF) [file ppat.1003150.s001.pdf]

Figure S2

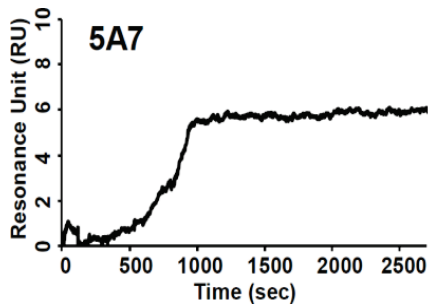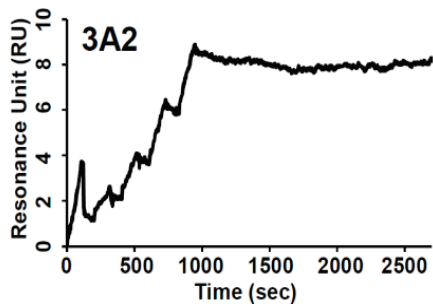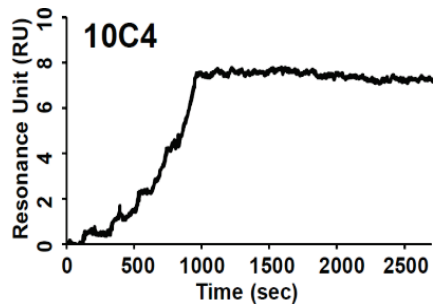

Supplement: Figure S2 — Surface plasmon resonance analysis of binding affinity between HuMAbs and the HA protein of B/Florida/4/2006. HuMAbs 5A7, 3A2 or 10C4 were immobilized on the surface of a CM5 chip via pre-crosslinked anti-human IgG Fcγ antibody. The HA protein at concentrations of 12.5, 25, 50, 100 and 200 nM were consecutively injected onto the chip surface and the association and dissociation phases were monitored. Signal from the chip surface without anti-HA antibody (the reference value) was subtracted from each reading. (PDF) [file ppat.1003150.s002.pdf]

Figure S3

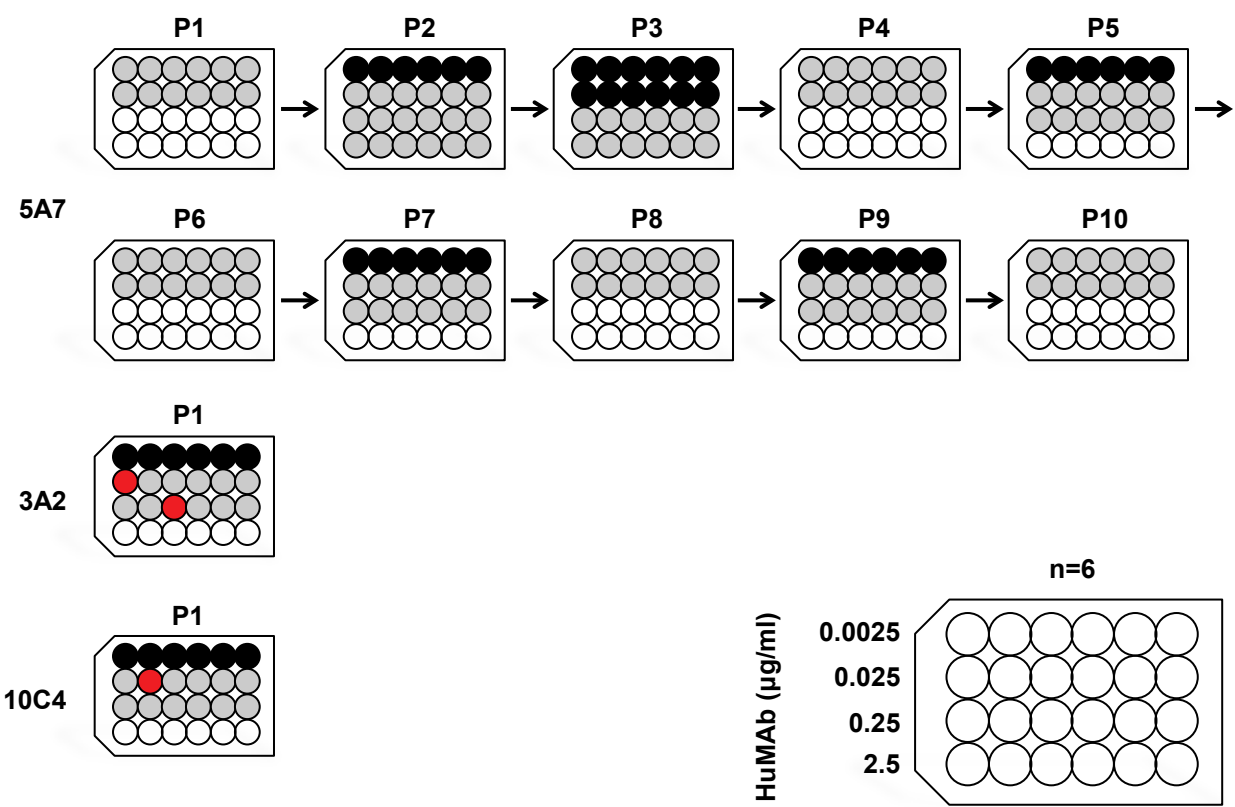

Supplement: Figure S3 — Diagram showing how the escape mutants were obtained. Four HuMAb concentrations prepared by serial ten-fold dilutions (right lower diagram) were mixed with B/Florida/4/2006 for 1 hour. Then, each mixture was used to infect MDCK cells in six wells (i.e., four groups of six wells) and incubated for 72 hours (for details, see Materials and methods). Groups were graded according to cytopathic effects: all six wells showing no cytopathic effects (white), some wells showing cytopathic effects (gray), and all wells showing cytopathic effects (black). Supernatants from wells colored gray were collected separately and measured for VN and HI activities. When a four-fold reduction in VN and HI assays was not shown by any of supernatants, one sample was mixed with HuMAbs serially 10-fold diluted described above and infected to newly prepared MDCK cells. P1 to P10 indicates passage number. Out of 12 gray wells, two wells for 3A2 and one well for 10C4 (colored red) showed a four-fold reduction in VN and HI activities compared with the parent virus. Gray wells at P10 of 5A7 and gray and red wells at P1 of 3A2 and 10C4 were subjected to direct sequencing analysis of the HA gene. (PDF) [file ppat.1003150.s003.pdf]

Figure S5

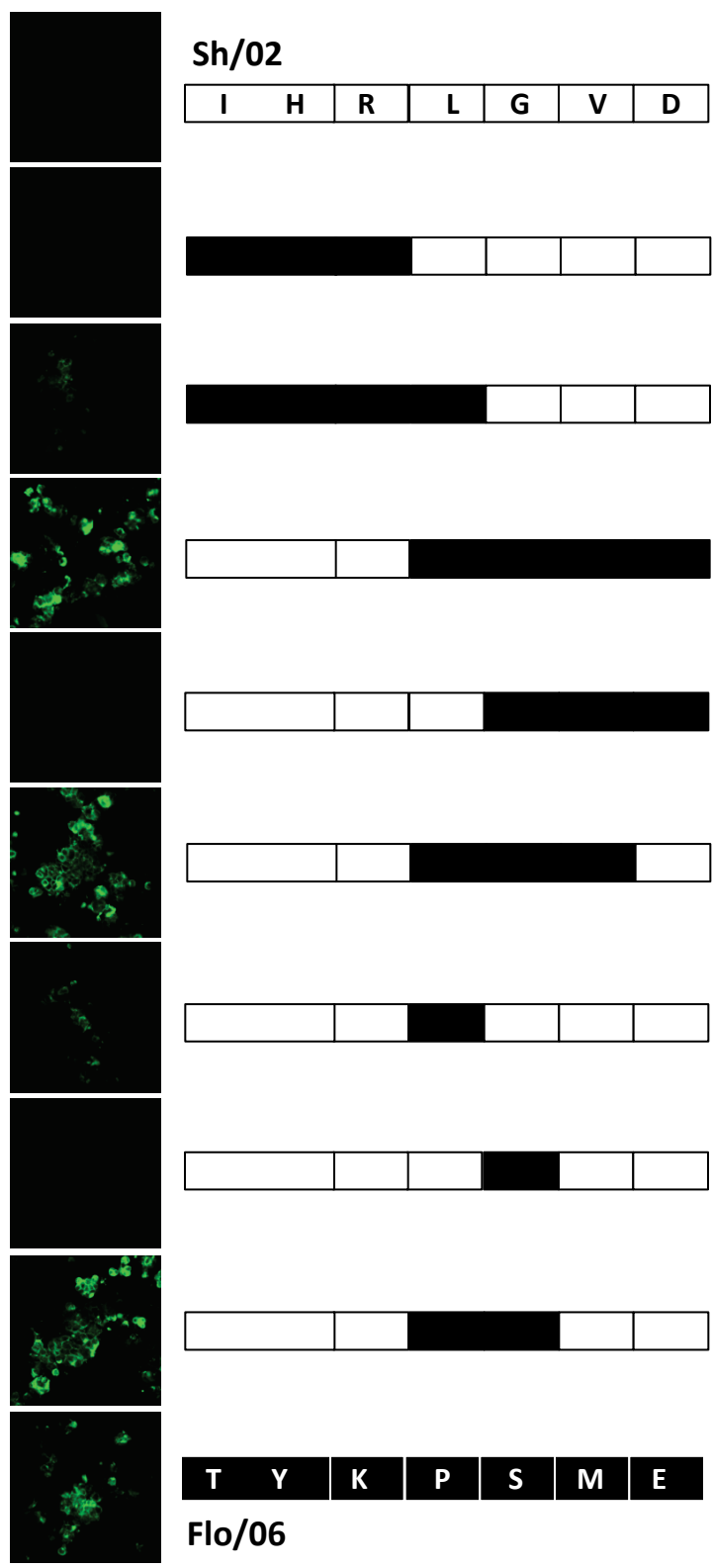

Supplement: Figure S5 — The additional epitope region of 3A2. Expression plasmids bearing chimeric HA protein were prepared from B/Shanghai/361/2002 (Sh/02) and B/Florida/4/2006 (Flo/06). 293T cells expressing the chimeric protein were subjected to IFA with 3A2 (left panels). White bars represent the amino acid sequence in Sh/02, and black bars represent the amino acid sequence in Flo/06. The different amino acid residues in the HA protein from each of the two viral strains are shown in the top and bottom bars. (PDF) [file ppat.1003150.s005.pdf]
