# Supplementary material for: Human Monoclonal Antibodies Broadly Neutralizing against Influenza B Virus
Source: PLoS Pathog. 2013 Feb 7;9(2):e1003150. doi: 10.1371/journal.ppat.1003150 (PMC3567173; doi:10.1371/journal.ppat.1003150)
Supplement: Figure S4 — The epitope region of 3A2 and 10C4. Escape mutants were selected by incubation of B/Florida/4/2006 with HuMAbs. Amino acid sequences of the HA protein in the escape mutants were compared with the original B/Florida/4/2006. Asterisks indicate amino acid residues that differed between the original virus and the escape mutants. (PDF) [file ppat.1003150.s004.pdf]

Figure S4

```
B_Florida_HA.gpt   1:DRICTGITSSNSPHVVKTATQGEVNVTGVIPLTTTPTKSYFANLKGTRTRGKLCPDCLNC 60
3A2_Esc.gpt         :-RICTGITSSNSPHVVKTATQGEVNVTGVIPLTTTPTKSYFANLKGTRTRGKLCPDCLNC
10C4_Esc.gpt        :DRICTGITSSNSPHVVKTATQGEVNVTGVIPLTTTPTKSYFANLKGTRTRGKLCPDCLNC

                  61:TDLDVALGRPMCVGTTPSAKASILHEVKPVTSGCFPIMHDRTKIRQLPNLLRGYENIRLS 120
                    :TDLDVALGRPMCVGTTPSAKASILHEVKPVTSGCFPIMHDRTKIRQLPNLLRGYENIRLS
                    :TDLDVALGRPMCVGTTPSAKASILHEVKPVTSGCFPIMHDRTKIRQLPNLLRGYENIRLS

                 121:TQNVIDAEKAPGGPYRLGTSGSCPNATSKSGFFATMAWAVPKDNNKNATNPLTVEVPYIC 178
                    :TQNVIDAEKAPGGPYRLGTSGSCPNATSKSGFFATMAWAVPKDNNKNATNPLTVEVPYIC
                    :TQNVIDAEKAPGGPYRLGTSGSCPNATSKSGFFATMAWAVPKDNNKNATNPLTVEVPYIC

                 179:TEGEDQITVWGFHSDDKTQMKNLYGDSNPQKFTSSANGVTTHYVSQIGSFPDQTEDGGLP 238
                    :TEGEDQITVWGFHSDNKIQMKNLYGDSNPQKFTSSANGVTTHYVSQIGSFPDQTEDGGLP
                    :TEGEDQITVWGFHSDNKNQMKNLYGDSNPQKFTSSANGVTTHYVSQIGSFPDQTEDGGLP
                                    * *
                 239:QSGRIVVDYMMQKPGKTGTIVYQRGVLLPQKVWCASGRSKVIKGSLPLIGEADCLHEKYG 298
                    :QSGRIVVDYMMQKPGKTGTIVYQRGVLLPQKVWCASGRSKVIKGSLPLIGEADCLHEKYG
                    :QSGRIVVDYMMQKPGKTGTIVYQRGVLLPQKVWCASGRSKVIKGSLPLIGEADCLHEKYG

                 299:GLNKSKPYYTGEHAKAIGNCPIWVKTPLKLANGTKYRPPAKLLKERGFFGAIAGFLEGGW 358
                    :GLNKSKPYYTGEHAKAIGNCPIWVKTPLKLANGTKYRPPAKLLKERGFFGAIAGFLEGGW
                    :GLNKSKPYYTGEHAKAIGNCPIWVKTPLKLANGTKYRPPAKLLKERGFFGAIAGFLEGGW

                 359:EGMIAGWHGYTSHGAHGVAVAADLKSTQEAINKITKNLNSLSELEVKNLQRLSGAMDELH 418
                    :EGMIAGWHGYTSHGAHGVAVAADLKSTQEAINKITKNLNSLSELEVKNLQRLSGAMDELH
                    :EGMIAGWHGYTSHGAHGVAVAADLKSTQEAINKITKNLNSLSELEVKNLQRLSGAMDELH

                 419:NEILELDEKVDDLRADTISSQIELAVLLSNEGIINSEDEHLLALERKLKKMLGPSAVEIG 478
                    :NEILELDEKVDDLRADTISSQIELAVLLSNEGIINSEDEHLLALERKLKKMLGPSAVEIG
                    :NEILELDEKVDDLRADTISSQIELAVLLSNEGIINSEDEHLLALERKLKKMLGPSAVEIG

                 479:NGCFETKHKCNQTCLDRIAAGTFNAGEFSLPTFDSLNITAASLNDDGLDNHTILLYYSTA 538
                    :NGCFETKHKCNQTCLDRIAAGTFNAGEFSLPTFDSLNITAASLNDDGLDNHTILLYYSTA
                    :NGCFETKHKCNQTCLDRIAAGTFNAGEFSLPTFDSLNITAASLNDDGLDNHTILLYYSTA

                 539:ASSLAVTLMLAIFIVYMVSRDNVSCSICL                                567
                    :ASSLAVTLMLAIF----------------
                    :ASSLAVTLMLAIF----------------
```
