# Supplementary material for: Human Monoclonal Antibodies Broadly Neutralizing against Influenza B Virus
Source: PLoS Pathog. 2013 Feb 7;9(2):e1003150. doi: 10.1371/journal.ppat.1003150 (PMC3567173; doi:10.1371/journal.ppat.1003150)
Supplement: Table S1 — Pattern of reactivity of HuMAbs. (PDF) [file ppat.1003150.s006.pdf]

Table S1. Pattern of reactivity of HuMAbs.

	Isotype	IFA[1]	Western blotting[1]	Target
5A7	IgG1	+[2]	+	HA of influenza B
3A2	IgG3	+	–[3]	HA of influenza B
10C4	IgG1	+	–	HA of influenza B

[1]MDCK cells infected with B/Florida/4/2006, homologous to vaccine antigen.

[2]Positive result.

[3]Negative result.
