# Supplementary material for: Human Monoclonal Antibodies Broadly Neutralizing against Influenza B Virus
Source: PLoS Pathog. 2013 Feb 7;9(2):e1003150. doi: 10.1371/journal.ppat.1003150 (PMC3567173; doi:10.1371/journal.ppat.1003150)
Supplement: Table S2 — Homology of the epitope region of HuMAb 5A7 among the corresponding sequences derived from NCBI database. (PDF) [file ppat.1003150.s007.pdf]

Table S2. Homology of the epitope region of HuMAb 5A7 among the corresponding sequences derived from NCBI database.

Sequence	Number of strains		Strain name	Accession number
	Yamagata lineage	Victoria lineage		
I**G**N**C**PI**W**VKT[1]	1427	1399		
V---------	0	5	B/California/03/2010,	AET21655,
			B/Florida/AF2746,	ADY16591,
			B/Florida/AF2747/2010,	ADY16592,
			B/Nairobi/2035/2006,	ABN71699,
			B/Niigata/10F554/2011	AEQ49737
--------R-	2	1	B/Hawaii/08/2009 (V)[2]	AER28587,
			B/Kisii/6531/2008 (Y),	ACM45921,
			B/Minnesota/08/2009 (Y)	AER28601
--D-------	1	2	B/Parma/1/2003 (V),	CAH56949,
			B/Florida/AF2750/2011 (Y),	ADY16607,
			B/Pucallpa/FLU5716/2007 (V)	ACC59730
L---------	2	0	B/Kol/1013/2007,	AEA51387,
			B/Kol/1266/2007	AEG21018
--------I-	2	0	B/Kol/583/2006,	AEA51386,
			B/Kol/667/2006	AEG21015
K-------N-	1	0	B/Kol/1230/2007	AEA51388
--H-------	1	0	B/Shanghai/361/2002	ABP52001
--I-------	0	1	B/Kol/N2121/2010	AEG21026
--T-------	0	1	Influenza B virus	AAA43692
--Y-------	0	1	B/Rio Grande do Sul/317/2008	ADG58671
---**F**------	1	0	B/Rio Grande do Sul/314/2008	ADG58665
---**W**------	0	1	B/Xuanwu/1/1982	AAK68702
-----T----	1	0	B/Pennsylvania/11/2007	ACA33432
-----V----	0	1	B/Lisboa/7/2002	ABY71796
--------Q-	0	1	B/Shanghai/35/1984	AAK68706
---------K	0	1	B/Managua/4485.05/2010	AEQ39337
---------P	1	0	B/Kol/1253/2007	AEA51390

[1]Critical amino acid residues for the epitope of 5A7 are shown in bold and underlined.
[2](V) is Victoria lineage and (Y) means Yamagata lineage.
